# Dual-Source Photon-Counting Computed Tomography—Part III: Clinical Overview of Vascular Applications beyond Cardiac and Neuro Imaging

**DOI:** 10.3390/jcm12113798

**Published:** 2023-05-31

**Authors:** Antonella Meloni, Filippo Cademartiri, Laura Pistoia, Giulia Degiorgi, Alberto Clemente, Carmelo De Gori, Vincenzo Positano, Simona Celi, Sergio Berti, Michele Emdin, Daniele Panetta, Luca Menichetti, Bruna Punzo, Carlo Cavaliere, Eduardo Bossone, Luca Saba, Riccardo Cau, Ludovico La Grutta, Erica Maffei

**Affiliations:** 1 Department of Radiology, Fondazione Monasterio/CNR, 56124 Pisa, Italy; antonella.meloni@ftgm.it (A.M.); laura.pistoia@ftgm.it (L.P.); giuliadegiorgi995@gmail.com (G.D.); clemente@ftgm.it (A.C.); degoricarmelo87@gmail.com (C.D.G.); positano@ftgm.it (V.P.); emaffei@ftgm.it (E.M.); 2 Department of Bioengineering, Fondazione Monasterio/CNR, 56124 Pisa, Italy; 3 BioCardioLab, Department of Bioengineering, Fondazione Monasterio/CNR, 54100 Massa, Italy; simona.celi@ftgm.it; 4 Cardiology Unit, Ospedale del Cuore, Fondazione Monasterio/CNR, 54100 Massa, Italy; sergio.berti@ftgm.it; 5 Department of Cardiology, Fondazione Monasterio/CNR, 56124 Pisa, Italy; emdin@ftgm.it; 6 Institute of Clinical Physiology, National Council of Research, 56124 Pisa, Italy; daniele.panetta@ifc.cnr.it (D.P.); luca.menichetti@ifc.cnr.it (L.M.); 7 Department of Radiology, IRCCS SynLab-SDN, 80131 Naples, Italy; bpunzo@sdn-napoli.it (B.P.); carlo.cavaliere@synlab.it (C.C.); 8 Department of Cardiology, Ospedale Cardarelli, 80131 Naples, Italy; ebossone@hotmail.com; 9 Department of Radiology, University Hospital, 09042 Monserrato, CA, Italy; lucasabamd@gmail.com (L.S.); riccardocau00@gmail.com (R.C.); 10 Department of Health Promotion, Mother and Child Care, Internal Medicine and Medical Specialties-ProMISE, Department of Radiology, University Hospital “P. Giaccone”, 90127 Palermo, Italy; lagruttaludovico@gmail.com

**Keywords:** photon-counting computed tomography, computed tomography angiography, vascular imaging, abdominal, thoracic

## Abstract

Photon-counting computed tomography (PCCT) is an emerging technology that is expected to radically change clinical CT imaging. PCCT offers several advantages over conventional CT, which can be combined to improve and expand the diagnostic possibilities of CT angiography. After a brief description of the PCCT technology and its main advantages we will discuss the new opportunities brought about by PCCT in the field of vascular imaging, while addressing promising future clinical scenarios.

## 1. Introduction

Computed tomography angiography (CTA) is actually considered the “gold” standard for the non-invasive assessment and diagnosis of vascular abnormalities [1,2]. CTA exploits the adequate opacification of the contrast of the target vessel following the intravenous administration of an iodinated contrast medium at high-flow rates [2]. The main advantages of CTA are the widespread availability, the short acquisition time, and the excellent spatial and temporal resolutions, while the main drawback is the exposure to ionizing radiation [1,2]. Moreover, the use of iodinated contrast media is not totally risk-free [3]. However, there is no doubt that CTA is far superior to invasive catheter-based angiographic techniques in terms of safety, costs, and even accuracy for most anatomical districts; and, even though CTA has been clinically implemented for several vascular territories, some limitations still persist and affect the reliability of the vascular assessment, especially when smaller arteries have to be assessed.

Current cutting-edge clinical CT systems are equipped with energy-integrating detectors (EIDs), which can provide images with quantitative information such as tissue-specific images and maps of the iodine concentration. With this technology, the spatial and contrast resolutions have been improving with quite small steps over the years. A different detection concept, which has gained increasing interest and is expected to substantially modify clinical CT imaging, is photon-counting detectors (PCDs). PCDs have multiple potential advantages over conventional EIDs, which can be combined to improve and expand the diagnostic possibilities of the CTA [4,5,6].

After a brief description of the photon-counting CT (PCCT) technology and its main advantages, this review will focus on its clinical applicability in the realm of vascular imaging, without considering the coronary and carotid districts. We will describe both the existing literature, which is still limited, and our current experience, with the aim of providing an idea of the potential clinical implementation and diagnostic impact. Therefore, several PCCT images acquired in our department will be provided as a proof of concept.

## 2. Photon-Counting CT Technology

**Figure 1:**
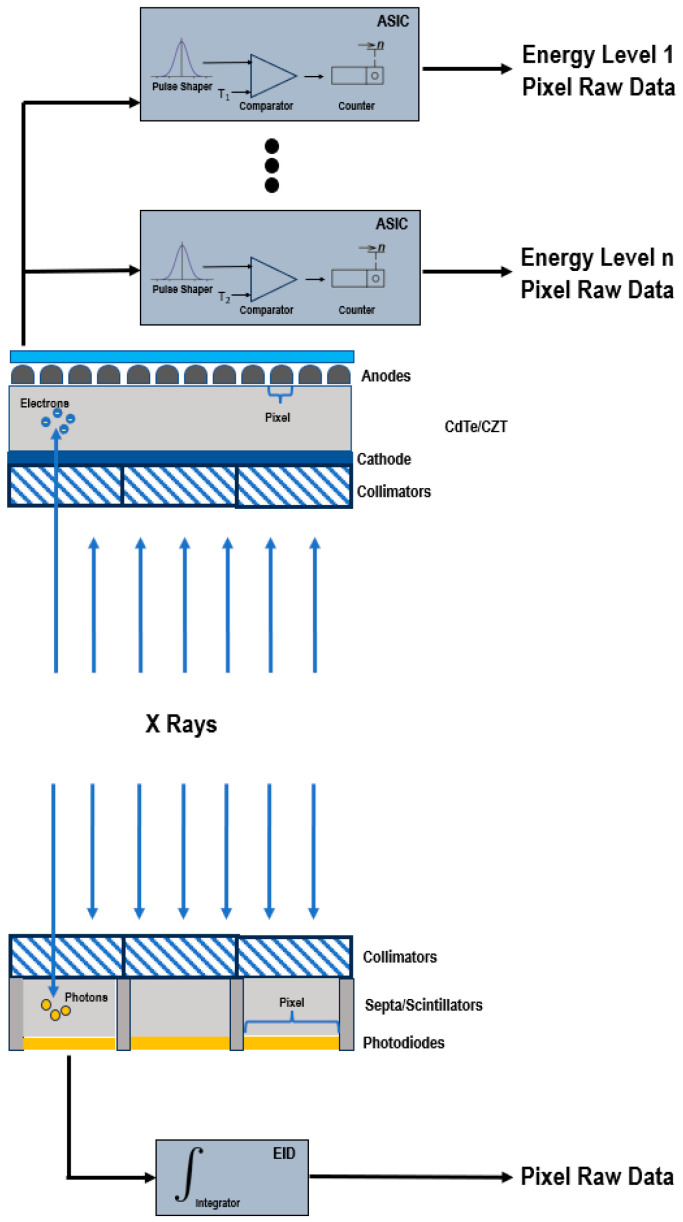
Schematic representation of a PCD (**top**) and of a conventional EID (**bottom**).

Figure 1 shows a schematic representation of EIDs and PCDs.

EIDs are composed of scintillator elements and septa. The scintillator converts the incident X-rays into visible light photons, that are then absorbed by a photodiode which produces an electrical signal proportional to the energy deposited and comprising also electronic noise [4]. The septa channel the light towards the sensor.

Instead, PCDs consist of a single thick layer of a semiconductor diode on which a large voltage is applied; incoming X-ray photons are directly converted into electronic signals [4,6,7]. The electrical signal is amplified and shaped, and its peak height is proportional to the energy of the individual X-ray photon.

Since PCDs do not require septa, there is no technical limitation for the pixel pitch and its size can be significantly reduced without negatively impacting the geometric detection efficiency. This translates into an increased spatial resolution, that can be exploited to generate sharper images and visualize finer details [7,8].

In conventional EIDs, the attenuation is dominated by the highest energies present in the X-ray tube spectrum. This affects the contrast-to-noise ratio (CNR), since the contrast between different materials is enhanced at low X-ray energies. Conversely, PCDs uniformly weight all photons and can provide a better signal to lower-energy photons, allowing for the improved signal of those substances that are highly attenuating at low energies, such as iodine and calcium, and the optimal CNR [9,10,11,12,13]. The energy-discriminating ability also represents the key to the elimination of the electronic noise achievable with PCDs. The electronics system of the detector counts how many pulses have a height exceeding a preset threshold level. By setting the lower threshold at a level higher than the electronic noise level, the signal resulting from the electronic noise, characterized by a low amplitude, is effectively discarded and only the pulses generated by incoming photons are counted. The lowest threshold energy is approximately 20 keV. Below this energy threshold, there are no X-rays in the primary beam of the X-ray tube, because they are removed by a prefilter.

The reduced electronic noise and the enhanced CNR and visualization of small objects contribute to the improvement of the dose efficiency [14,15], promoting the development of new low-dose CT acquisitions. Comparison studies between PCCT and conventional CT demonstrated that PCCT allowed for scanning with a lower radiation dose (43–50% reduction) while retaining a similar or better objective or subjective image quality [16,17,18]. Moreover, not only may be the radiation dose potentially decreased, but also the iodine contrast concentrations, which may be particularly beneficial for patients with decreased kidney function.

In addition to the weighting of energy, the other primary mechanism for employing the energy information from spectral PCCT data is material decomposition. The application of algorithms for material decomposition from a number of energy-selective images generates a set of basis image maps. The number of bases depends on the number of spectral data (N bases for N spectral data) and can be increased to N+1 by imposing mass or volume conservation constraints, which, if not correct, can cause inaccuracies [19]. Each base image map contains the equivalent material concentration displayed through a voxel-by-voxel approach. The basis material images can be displayed directly, revealing the distribution of a certain material, such as a contrast agent, or they can be processed to obtain virtual monochromatic images (VMI) [20,21,22], virtual non-contrast images [23], or material-specific color-overlay images [24]. Since EID-based CT acquires data in two energy regimes, it can accurately separate, without assumptions, one contrast agent, e.g., iodine, from the background, but it is not able to discriminate between two contrast materials with a high atomic number (high Z). PCDs differentiate photons of different energies based on the pulse-height and enable simultaneous multi-energy (N ≥ 2) acquisitions with supreme spatial and temporal registrations and without spectral overlap [25]. The increase in the number of energy regimens improves the precision in the measurement of each photon energy, resulting in better material-specific or weighted images [26] and improved quantitative imaging. Indeed, the concentrations of materials, such as contrast agents and calcium, can be quantitatively assessed, independent of acquisition parameters. Another benefit associated with the use of multiple energy measurements is the possibility to quantify elements with a K-edge in the diagnostic energy range and, consequently, to employ alternative contrast agents from iodine, such as gold, platinum, silver, bismuth, or ytterbium [27,28,29,30], or to design new types of contrast agents such as nanoparticles targeted at specific cells or enzymes [31,32,33,34]. This unique opportunity clears the way for molecular and functional CT imaging and for the simultaneous multi-contrast agent imaging. As clearly demonstrated in animal or proof-of-concept (in silico) studies, PCDs allows for the clear differentiation of more than two contrast agents in each voxel at the time of acquisition, with different pharmacokinetics within the same biological system [24,35,36,37]. For each component, it is possible to generate separate quantitative maps, showing its specific distribution, which may potentially result in additional clinical information.

Another important benefit of PCCT is the reduction of common image artifacts. The elimination of the electronic noise allows for a marked reduction of the streak and shading artifacts [13]. The constant weighting decreases the beam-hardening artifacts [38,39]. In particular, the high-energy bin image offers the highest advantages in the improved immunity to beam-hardening effects [40,41]. PCDs reduce metal and calcium blooming as a result of the improved spatial resolution (reduction in voxel size and partial voluming) and the improved material decomposition [42].

## 3. Vascular Applications of PCCT beyond Cardiac and Neuro Imaging

Table 1 summarizes the previous studies on PCCT in vascular imaging, excluding the coronary and carotid districts.

**Figure 2:**
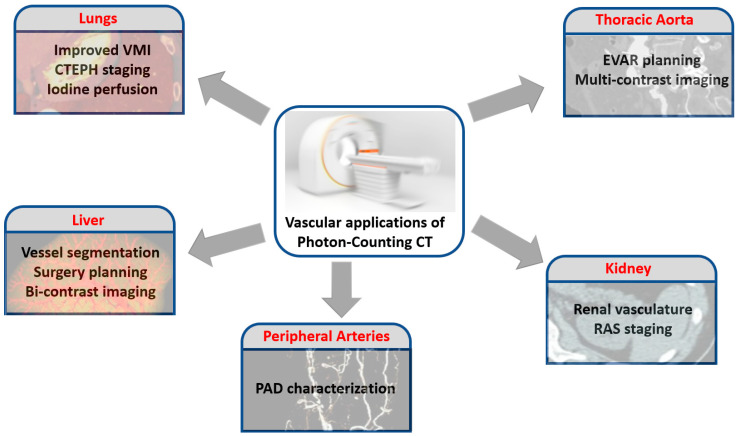
Potential applications of PCCT in the realm of the vascular imaging, without considering the coronary and carotid districts.

Figure 2 summarizes some of the potential vascular applications of PCCT.

### 3.1. Pulmonary Vascularization

Pulmonary vascular diseases encompass a broad and heterogeneous group of conditions including acute and chronic pulmonary thromboembolism, pulmonary hypertension, congenital abnormalities, and inflammatory vasculitis.

Pulmonary embolism (PE), that is the blockage of one of the pulmonary arteries caused by thromboembolic material, is associated with high morbidity and mortality [52].

**Figure 3:**
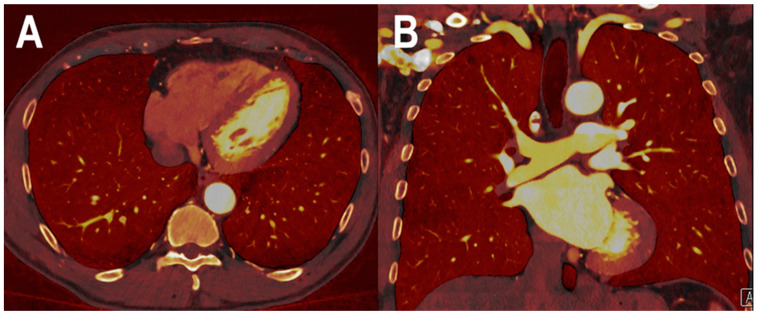
PCCT spectral iodine perfusion map of the lung parenchyma. The figure shows a PCCT spectral iodine perfusion map of the lung parenchyma acquired with a routine ECG-gated spiral acquisition through the entire chest in the arterial phase ((**A**) axial; and (**B**) coronal). The homogeneous distribution of the iodine map in all sectors represents a normal arterial perfusion of the lung parenchyma.

Multi-detector computed tomography permits the rapid accurate exclusion and diagnosis of PE and has become the imaging modality of choice for these purposes [53,54]. Dual-energy computed tomography (DECT), allowing the reconstruction of VMI at arbitrary energy levels (in keV), offers the advantage of providing both morphological and functional data. Indeed, the iodine maps accurately depict the consequences of PE in terms of parenchymal perfusion, improving the detection of small occluding emboli and enabling a quantitative analysis of the perfusion defect volume [55,56,57]. The extent of perfusion defects was demonstrated to correlate with adverse clinical outcomes, allowing the identification of high-risk patients requiring intensified monitoring and treatment [58]. Figure 3 shows a PCCT iodine perfusion map of the lung parenchyma. In phantom studies, PCCT demonstrated promising results in terms of VMI reconstruction. It showed accuracy in iodine quantification and a VMI CT number similar to that obtained with EID-based CT scanners, while offering a perfect temporal and spatial alignment to prevent motion artifacts, high spatial resolution, and the improvement of image noise and CNR [21,59]. Therefore, VMI obtained with a PCCT may improve lesion detection and promote the reduction of the contrast agent and radiation dose. Yalynska et al., evaluated in vivo the impact of different VMI energies in the diagnosis of PE with PCCT and detected the lowest levels of pulmonary artery attenuation and image noise corresponding with the highest-evaluated VMI energy (70 keV). Moreover, although the highest SNR was obtained with the lowest VMI (40 keV), the best subjective PE visibility was achieved at 50 keV, almost certainly due to the decreased image noise and number of hardening artifacts [43]. Importantly, with PCCT, the usage of low monoenergetic images would allow us to foster iodine attenuation, helping to decrease the amount of iodinated contrast agent for a pulmonary CT angiography.

In the study from Kopp et al., a custom-made lung phantom and a rabbit lung were imaged with a preclinical PCCT and a conventional CT in standard and high-resolution (HR-CT) modes [44]. According to the opinion of one experienced radiologist, the PCCT images showed a superior image quality in terms of spatial resolution and visibility of lung vessels compared to the HR-CT images.

Hagen et al. scanned one hundred consecutive oncologic patients with both PCCT and DECT and demonstrated that the radiation dose for the contrast-enhanced chest CT could be remarkably decreased (43% dose reduction based on size-specific dose estimates) with PCCT without scarifying the image quality. Combining the measurements of pulmonary vessels and the aorta resulted in a significantly higher CNR for PCCT compared to DECT [18].

The improved performance of PCCT may open the doors for a better detection and quantification of the changes in the proximal and distal pulmonary vascular tree. The accurate monitoring of the distal pulmonary vascular involvement is of particular interest in the setting of the chronic thromboembolic pulmonary hypertension (CTEPH) and of coronavirus disease 2019 (COVID-19). CTEPH, currently classified as a group 4 pulmonary hypertension (PH), is a rare but progressive disease, requiring timely diagnosis and treatment [60]. CTEPH involves not only persistent thrombi in proximal pulmonary arteries, but also a microvasculopathy which is crucial for the development and progression of the disease, contributing to severe hemodynamics [61]. The pulmonary vasculature is a possible target in COVID-19 and several mechanisms are involved in the pathophysiology of the damage [62]. Microvasculopathy with the formation of microthrombi plays a role in the COVID-19-related hypoxemia [63]. Thus, the early and accurate recognition of the pulmonary vascular signs may help to improve the outcome of several COVID-19 patients.

A study performed on 60 patients showed that a gadolinium chelate could be used as an alternative contrast agent for the CT examination of the pulmonary vasculature in patients for whom the administration of iodinated contrast material is contraindicated. The vascular enhancement was good/excellent and provided diagnostic information in 92% of the patients [64]. However, the attenuation values in the pulmonary vessels were much lower in comparison to that measured in typical iodine-based CT examinations (350–400 HU). Gadolinium is an ideal candidate for K-edge CT imaging, not feasible with DECT. Since the threshold settings for energy bins on both sides of a K-edge strongly influence the spectral image quality with regard to the contrast and noise level, the optimal partition of the two energy bins around a K-edge is critical. Several approaches have been introduced to optimize the energy bin width [65,66]. It may be expected that K-edge imaging by spectral CT may allow us to enhance the overall image quality (signal-to-noise ratio and contrast-to-noise ratio) and the diagnostic value of gadolinium-enhanced CT angiograms.

### 3.2. Hepatic Vasculature

The accurate and robust definition of hepatic vascular structures, known as liver vessel segmentation, is critical for the diagnosis and the radio-frequency ablation and surgical treatments of liver diseases [67], and plays an important role in liver transplant programs, for the accurate selection of potential donors [68]. Due to the high variability of the liver and vascular anatomy and the generally low contrast between vessels and the surrounding tissue, liver segmentation in conventional contrast-enhanced liver CT images remains challenging [69]. Baek et al. demonstrated that PCCT, enhancing the contrast of iodine-injected vascular structures in the liver, allowed for accurate liver vessel segmentation [46]. They proposed a deep-learning approach for liver vessel segmentation and used it for both PCCT and conventional CT images, generated with abdominal simulation phantoms, iodine-enhanced for the liver blood vessels. The PCD-CT datasets were created by using the multi-energy information. The segmentation using PCD-CT outperformed the one using conventional CT in terms of both the dice overlap score and 3D vascular structure visualization, and, in many cases, allowed to avoid the incorrect segmentation of the peripheral part of the vessels.

Si-Mohamed et al., evaluated in vivo in adult rats the capability of PCCT with high spatial resolution to discriminate between two contrast agents: gadolinium and iodine, which were administered simultaneously, one with an intraperitoneal injection and the other one with intravenous injections [45]. A clear visual separation of the contrast agents was present in the contrast material maps, with organs and vessels well-delineated. Importantly, very small structures such as the hepatic veins and the mesenteric vessels were detectable all along their way.

### 3.3. Thoraco-Abdominal Aorta

The endovascular aneurysm repair (EVAR) with a stent graft represents a valid alternative to open surgery for the treatment of abdominal aortic aneurysms [70,71]. However, in 25% of patients, EVAR is complicated by endoleaks [72]. CT angiography represents the standard of reference in both pre-operative planning and post-procedural surveillance [73]. Despite the undeniable benefits of triphasic CT scanning, the associated radiation exposure is inherently high compared to single-phase CT examinations [74]. Although a single-phase, dual-energy CT with a split-bolus technique and the reconstruction of virtual non-enhanced images can significantly reduce the radiation dose without having an impact on the endoleak detection rate [75], it does not allow the precise determination of the type of endoleak (either low- or high-flow). Dangelmaier et al., demonstrated in an abdominal aortic aneurysm phantom the capability of PCCT in combination with a dual-contrast agent injection (gadolinium and iodine) to replace multi-phase CT scans in the native, arterial, and delayed phase to efficiently capture endoleak dynamics [47]. The derived material maps could differentiate iodine, gadolinium, and calcium, enabling the reliable distinction of leaking contrast media and intra-aneurysmatic calcifications in a single scan, with a significant reduction of radiation exposure. With regard to the imaging of multiple contrast agents, Symons et al. demonstrated in vitro and in vivo in a large animal model the possibility of using PCCT with four energy bins to separate three contrast agents (gadolinium, iodine, and bismuth) [24]. Time-attenuation curves for gadolinium and iodine, in the regions of interest delineated in the aorta, showed a clear differentiation of the contrast materials.

Sigovan et al., demonstrated the ability of PCCT to improve vascular imaging in the presence of metallic stents [76]. Stents composed of different metals, positioned inside plastic tubes containing hydroxyapatite spheres simulating vascular calcifications and in the abdominal aorta of one New Zealand white rabbit, were imaged with a PCCT prototype, a dual-energy CT system, and a 64-channel CT system. Compared to the other CT systems, PCCT led to a superior lumen delineation and visualization of the stent’s metallic mesh, by virtue of the enhanced spectral and spatial resolution which allowed for a significant reduction of blooming artifacts. Moreover, with PCCT, the platinum-specific K-edge imaging allowed for the exclusive visualization of the stent made of platinum and the elimination of other backgrounds and contrast media.

The study of Higashigaito et al. was the first to assess the quality of contrast-enhanced abdominal PCCT acquired in a clinical setting [48]. Thirty-nine patients underwent both PCCT and EID-based CT with the same contrast media protocol. In PCCT, VMI were reconstructed in 10 keV intervals (40–90 keV). At the equivalent radiation dose, clinical PCCT with the reconstruction of VMI at 40, 50, and 60 keV showed the significantly higher CNR of vascular structures (abdominal aorta, inferior cava vein, and portal vein) compared to conventional EID-CT. This improvement may be used to further optimize the radiation dose and/or contrast media volume. In accordance with this study, Euler et al. showed in a cohort of forty patients that PCCT angiography of the thoraco-abdominal aorta with VMI at 40 and 45 keV led to a significantly increase in CNR compared with EID-CT at an identical radiation dose [49]. The CNR gain of PCCT was 34% higher for overweight patients compared with normal-weight patients. Very recently, the same group published a study involving 100 patients scanned with both EID-CT and PCCT and divided into two groups [50]. In the first group of 40 patients, where the same contrast media protocol was adopted for both scans, PCCT of the thoracoabdominal aorta using VMI at 50 keV provided, compared with EID-CT, the best trade-off between CNR (25% increase) and overall subjective image quality, which took into account image noise, vessel attenuation, and vessel sharpness. In the second group of 60 patients, the contrast media volume during the PCCT scan was reduced by 25%. No difference in the CNR and subjective image quality between EID-CT and PCCT at 50 keV with a reduced contrast media was found, demonstrating that the gain of image quality achievable with PCCT could be effectively translated into a low-volume contrast media protocol.

**Figure 4:**
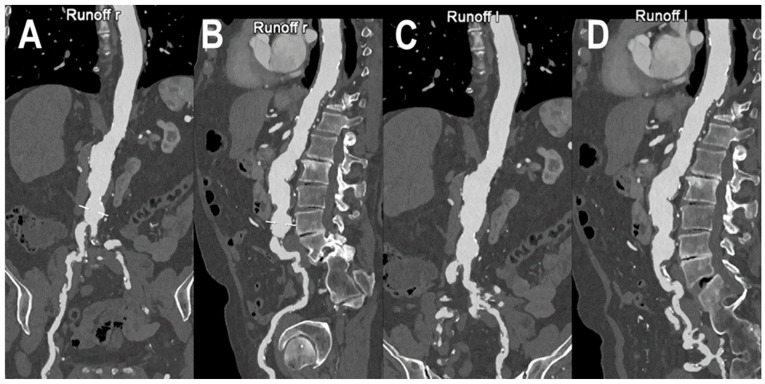
PCCT angiography of abdominal aorta. PCCT angiography of abdominal aorta showing the severe atherosclerotic alterations of the infra-renal section with multiple aneurysmatic segments until the carrefour (**A**–**D**). In (**A**,**B**) the orthogonal longitudinal curved MPR shows the aorta and the right ileo-femoral arterial axis, while, in (**C**,**D**) it shows the aorta and the left ileo-femoral arterial axis. Calcifications are spread throughout the aortic wall and the ileo-femoral axes with no impact on the capability of assessment of the lumen reduction due to the atherosclerotic alterations.

**Figure 5:**
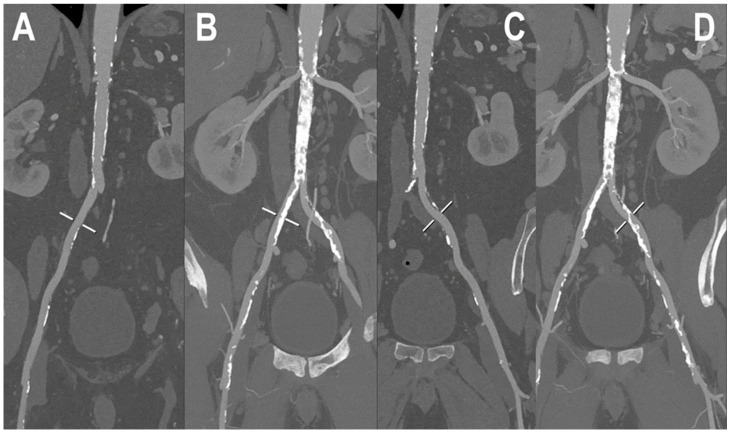
PCCT angiography of abdominal arteries. PCCT angiography of the abdominal arteries in a patient with severe calcifications of the arterial walls both at the level of the aorta and at the level of common iliac arteries (**A**–**D**). While MIP curved longitudinal reconstructions show extensive severe calcifications (**B**,**D**) which may hamper lumen assessment, in (**A**,**C**) the visualized segments are perfectly assessable, regardless of the amount of arterial wall calcifications. Renal arteries appear to be very wide and patent bilaterally (**B**,**D**).

A PCCT angiography of abdominal aorta is displayed in Figure 4 while Figure 5 shows a PCCT angiography of abdominal arteries in a patient with severe calcifications of the arterial walls at the level of the aorta.

### 3.4. Renal Vasculature

Volume-rendered CTA has become the key modality for the non-invasive assessment of conditions affecting the renal vasculature, including both arterial disorders such as renal artery stenosis, renal artery aneurysms, and dissection, as well as venous disorders such as thrombosis, splenorenal shunts, and intravascular tumor extension [77]. Moreover, CTA accurately displays the changes in the renal vascular structures, which should be detected before any type of renal surgery or interventional radiologic procedure [78].

The inherent advantages of PCCT could be particularly beneficial also in the evaluation of the renal vasculature. First of all, the enabled dose-efficient high-spatial-resolution imaging may impact several diagnostic pathways, such as the detection of renal artery stenosis (RAS) in children. Indeed, the decreased caliber of the renal arteries in children compared to adults and the frequent second- and third-order branch stenosis may affect the utility and performance of CTA in the diagnosis of pediatric RSA [79]. Otherwise, it is largely appreciated that aneurysms secondary to small-vessel vasculitis are difficult to identify in CTA, due to the limited resolution. A technical feasibility study tested a high-resolution PCCT protocol (0.25 mm at the isocenter) in phantom, animals, and eight human subjects, and demonstrated an improved spatial resolution and reduced image noise and, above all, a better visualization of distal vessels in the lung images [51].

Another high-impact clinical benefit of PCCT is represented by its multi-energy capability. In RAS, as well as in coronary artery disease, the fibrous tissue is the most prominent plaque composition, followed by fibro-fatty, necrotic core, and dense calcium tissue [80]. The absence of predictive indices for the favorable patient and lesion selection conditions the clinical benefits associated with the percutaneous revascularization of atherosclerotic RAS [81]. The accurate evaluation of the RAS plaque composition may enable the identification of those lesions more likely to result in embolization during stenting [82]. In this context, the current limitations associated with the conventional CT may be overcome by the multi-energy PCCT imaging combined with nanoparticles loaded with novel CT contrast agents. In particular, the gold nanoparticles designed for uptake by monocytes and macrophages have been demonstrated, in animal studies, to accumulate in atherosclerotic plaque [83]. Cormode et al. demonstrated in a phantom of an artery the ability of PCCT to accurately discriminate between the gold nanoparticle contrast agent, iodine-based contrast agent, and calcium-rich material [32], thus showing the potential for the complex pathophysiologic characterization of plaque.

### 3.5. Peripheral Arterial Disease

Peripheral artery disease (PAD) is the partial or complete obstruction of any part of the peripheral arterial tree, mainly caused by arteriosclerosis [84]. CTA is the preferred image modality for the cross-sectional imaging of PAD since it allows us to delineate the arterial tree and to determine the amount of atherosclerotic burden [85]. However, the diagnostic accuracy may be compromised by the presence of dense calcifications [85,86] which can cause blooming and partial volume artifacts. These artefacts can result in an overestimation of stenotic disease [87,88]. Moreover, the attenuation properties may be comparable between calcifications and the iodinated lumen.

PCCT, with its intrinsic possibility of reducing blooming by means of improved spatial resolution and material decomposition, may allow for a more detailed and non-invasive characterization of PAD. Li et al., developed a method for quantifying the percent area of stenosis based on the material decomposition of dual-energy and multiple-energy CT images, without the need of segmentation [89]. The ability of this method to reduce the partial volume and blooming effects was proven by computer simulations. The experiments, conducted on phantoms with different stenosis severity, vessel diameters, and calcification densities, showed more accurate and precise stenosis measurements for four-threshold PCCT images than for DECT and two-threshold PCCT images. In addition, the three-basis-material decomposition made directly on four-threshold PCCT images permitted to produce calcium, iodine, and water image maps.

A recent review highlighted as the combination of ultra-high-resolution CT (UHRCT) with advanced post-processing techniques, such as subtraction techniques, has allowed us to reduce the impact of calcium blooming [90]. However, this approach has not been fully validated at this moment and comes with a reduction of dose efficiency. The UHR imaging technique has been implemented also on PCD-based CT systems [91]. A study performed on anthropomorphic phantoms and cadaveric specimens has demonstrated a 29% reduction of noise in UHR PCD images compared to the UHR EID images, which can be translated in a potential dose savings of 50% for identical image noise [91].

At present, there is no systematic study demonstrating in vivo the performance of PCCT for noninvasive imaging and the treatment planning of peripheral arterial disease.

**Figure 6:**
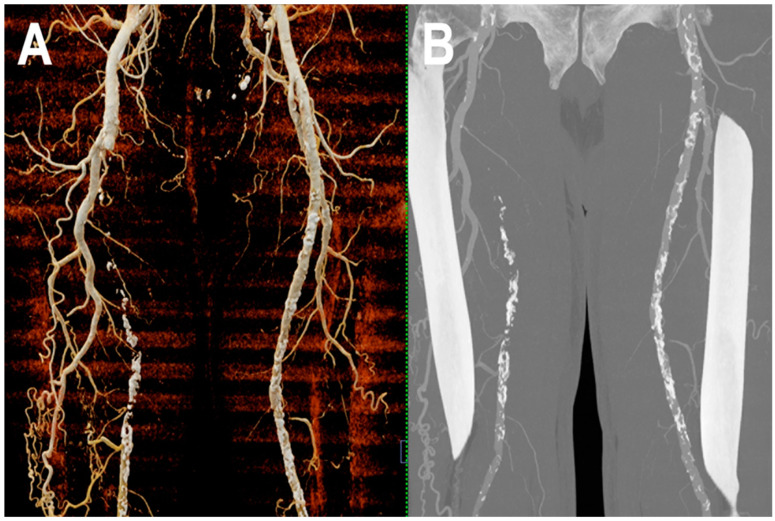
PCCT angiography of superficial femoral arteries. Cinematic rendering (**A**) and thin-MIP (**B**) PCCT angiography of superficial femoral arteries with severe and diffuse atherosclerotic calcified plaques bilaterally. The proximal part and the middle third of the right superficial femoral artery is occluded with wide collateralization through the right deep femoral artery. Very thin collateral arteries are easy to visualize on post-processed images.

**Figure 7:**
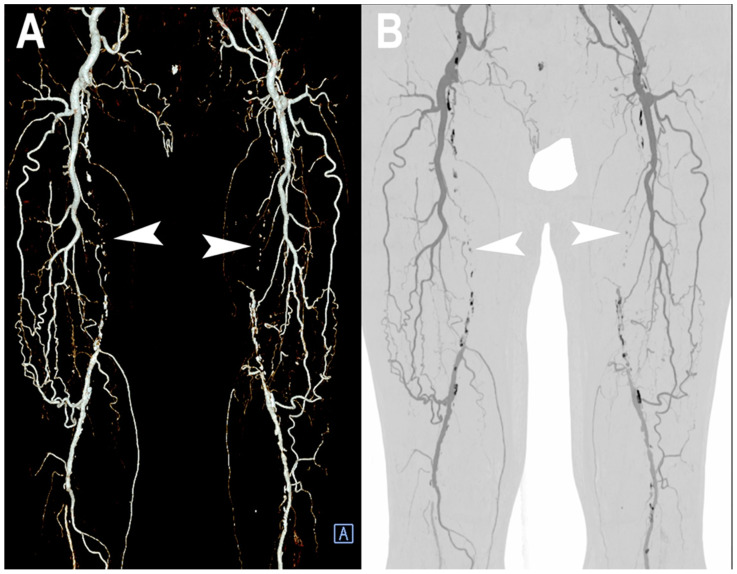
PCCT angiography of superficial femoral arteries. 3D volume rendering (**A**) and thin-MIP (**B**) PCCT angiography of superficial femoral arteries with severe and diffuse atherosclerotic calcified plaques bilaterally (**A**,**B**). The proximal part and middle third of the superficial femoral arteries is occluded bilaterally ((**A**,**B**): arrowheads) with wide collateralization through the deep femoral arteries. Very thin collateral arteries are easy to visualize on post-processed images.

**Figure 8:**
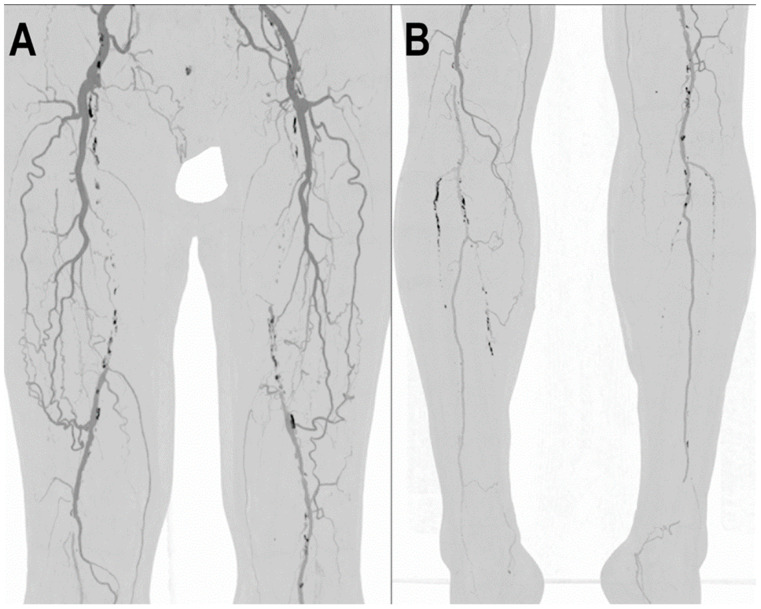
PCCT angiography of lower limbs. PCCT angiography of the lower limbs displayed with volumetric MIP post-processing in angio-mode (**A**,**B**). In A, there is a bilateral total chronic occlusion of the proximal and middle third of the superficial femoral arteries with wide bilateral collateralization through branches of the deep femoral arteries. In (**B**), there is a bilateral total chronic occlusion of the tibialis anterior and posterior; relatively preserved is the flow and patency of both interosseous arteries.

Figure 6 and Figure 7 show a PCCT angiography of superficial femoral arteries while Figure 8 shows a PCCT angiography of the lower limbs.

## 4. Conclusions

The key benefits of PCCT, such as improved spatial resolution, signal and contrast behavior, significant noise reduction, dose efficiency, and multi-energy capability, may provide the opportunity to further enhance the diagnostic and prognostic value of CTA. In any case, more extensive research is needed to demonstrate how the theoretical and proven advantages of PCDs can be transferred into clinical practice.

## Figures and Tables

**Table 1:** Studies on PCCT for vascular applications beyond cardiac and neuro imaging.

Authors	Years	Model	Number of Patients	Clinical Application	Results
Yalinska et al. [43]	2022	In vivo (human)	8	Pulmonary vascularization	In multi-energy PCCT images, the best signal was achieved at the lowest VMI (40 keV), while the best visualization of the pulmonary embolism was obtained at 50 keV, due to decreased image noise and hardening artefacts.
Kopp et al. [44]	2022	In vitro		Pulmonary vascularization	Compared to conventional and HR CT images, PCCT offered an increased spatial resolution and better visibility of lung vessels.
Hagen et al. [18]	2022	In vivo (human)	100	Pulmonary vascularization	Combining vessel measurements of the aorta and pulmonary vessels resulted in a significantly higher CNR of PCCT compared to EID-CT.
Si-Mohamed et al. [45]	2018	In vivo (animal)		Hepatic vasculature	Very small structures such as the hepatic veins and the mesenteric vessels were accurately detected all along their way with PCCT.
Baek et al. [46]	2022	Simulation		Hepatic vasculature	PCCT outperformed the standard deep-learning segmentation method with conventional CT in terms of 3D vascular structure visualization.
Symons et al. [24]	2017	In vitro and in vivo (animal)		Aorta	A simultaneous material decomposition of three contrast agents in vivo (gadolinium, iodine, and bismuth) was possible with PCCT.
Dangelmaier et al. [47]	2018	In vitro		Aorta	PCCT in combination with a dual-contrast agent injection was able to capture endoleak dynamics and the obtained material maps could differentiate iodine, gadolinium, and calcium.
Higashigaito et al. [48]	2022	In vivo (human)	39	Aorta	PCCT with reconstructions of VMI at 40, 50, and 60 keV showed a significantly increased CNR at similar image quality compared to EID-CT at identical radiation dose.
Euler et al. [49]	2022	In vivo (human)	40	Aorta	Compared with EID-CT at matched radiation dose, PCCT angiography of the aorta with VMI at 40 and 45 keV resulted in a significant increase of CNR. The CNR gain was higher in overweight patients.
Higashigaito et al. [50]	2023	In vivo (human)	100	Aorta	Angiography of the aorta with PCCT was associated with higher CNR compared to conventional CT. PCCT with a low-volume contrast media protocol gave comparable image quality to that of EID-CT at the same radiation dose.
Pourmorteza et al. [51]	2018	In vitro and in vivo (animal and human)	8	Renal vasculature	High-resolution PCCT improved the visualization of distal vessels in lung images.

## Data Availability

Not applicable.
